# Long non-coding RNA RNF7 promotes the cardiac fibrosis in rat model via miR-543/THBS1 axis and TGFβ1 activation

**DOI:** 10.18632/aging.102463

**Published:** 2020-01-08

**Authors:** Fan Ouyang, Xiangyang Liu, Guoan Liu, Haihua Qiu, Yi He, Hongyu Hu, Ping Jiang

**Affiliations:** 1 Department of Cardiology, Zhuzhou Hospital, The Affiliated Hospital of Xiangya Medical College of Central South University, Changsha, Hunan, China

**Keywords:** cardiac fibrosis (CF), TGFβ1, TSP-1, lncRNA RNF7, miR-543

## Abstract

Cardiac fibrosis (CF) is regulated by multiple factors, including transforming growth factor β1 (TGFβ1) and non-coding RNAs. Thrombospondin 1 (TSP1) is a physiologic regulator of TGFβ activation. Here, we performed microarray analyses on mRNAs and lncRNAs differentially-expressed in the CF and normal rat hearts. KEGG signaling annotation and GO enrichment analyses were performed to validate the roles of extracellular matrix (ECM) and TSP1-enhanced TGFβ activation in CF. The co-expression network between differentially-expressed lncRNAs and ECM-related factors was constructed to identify candidate lncRNAs and miRNAs. We found that lncRNA Homo sapiens ring finger protein 7 (lnc RNF7) was significantly correlated with TSP1 and ECM. Lnc RNF7 silence could attenuate isoproterenol (ISP)-induced CF in rat heart *in vivo* and in rat cardiac fibroblasts *in vitro*. Moreover, angiotensin II (Ang II) -induced CF in rat cardiac fibroblasts could also be attenuated by Lnc RNF7 silence. Furthermore, miR-543 could simultaneously target lnc RNF7 and 3' UTR of TSP1. Lnc RNF7 silence suppressed, while miR-543 inhibition promoted TSP1 protein and TGFβ activation, as well as ECM markers expression. The effects of lnc RNF7 silence was significantly reversed by miR-543 inhibition. In conclusion, CF progression might be regulated by lnc RNF7/miR-543 axis via TSP1-mediated TGFβ activation.

## INTRODUCTION

Cardiac fibrosis (CF) is characterized by increased activity of cardiac fibroblasts and the production of excessive extracellular matrix (ECM) in the myocardium [1]. Fibrosis results in cardiac stiffness, thereby compromising cardiac output and eventually leading to heart failure [2]. Since fibrosis plays a central role in the pathology of cardiac diseases, understanding of this process is helpful in identifying therapeutic targets.

CF is regulated by multiple factors, including transforming growth factor β1 (TGFβ1) [3, 4], angiotensin II (Ang II) [5], endothelin-1 [6], and platelet-derived growth factor [7], among which TGFβ1 is one of the most important. TGFβ is produced and secreted by platelets, leukocytes, and fibroblasts in the infarcted myocardium [8]. TGFβ1 induces myofibroblast transdifferentiation and enhances ECM protein expression [9, 10]. TGFβ is synthesized as an inactive latent complex. A significant regulatory step in determining appropriate levels of TGFβ is bioactivation of the latent complex [11]. The matricellular protein Thrombospondin 1 (TSP1) is a physiologic regulator of latent TGFβ activation *in vitro* and in many homeostatic and pathologic conditions *in vivo* [12–15]. The increases in TSP1 expression result in enhanced TGFβ activation and increased synthesis of ECM proteins. These data suggest that TSP1 plays a vital role in the development of fibrosis via enhancing TGFβ activation.

Non-coding RNAs are involved in the regulation of CF. Several long noncoding RNAs (lncRNAs), which are longer than 200 nucleotides and not translated into proteins, are dysregulated in patients with CF. By acting as competing endogenous RNAs (ceRNAs), lncRNAs post-transcriptionally regulate microRNA (miRNA) levels by homologous base pairing, therefore modulating mRNA stability and translation [16]. As for miRNAs, they could function either to promote (miR-21, miR-34, miR-199b, and miR-208) or to inhibit (miR-1, miR-26a, miR-29, miR-101, miR-122, miR-133/miR-30, miR-133a, and miR-214) CF [17]. LncRNA cardiac hypertrophy-related factor (CHRF) plays a vital role in cardiac hypertrophy through its sponge-like action on miR-489 [18]. LncRNA cardiac apoptosis-related lncRNA (CARL), regulates apoptosis by targeting miR-539 and PHB2 in mice with myocardial infarction [19]. Evidence for the role of ncRNAs regulation of gene expression in the development of CF has been developed. Thus, we hypothesize that lncRNA(s) and miRNA(s) may form a regulatory axis to modulate CF via TSP1-enhanced TGFβ activation. However, analysis on ncRNAs remains challenging since lncRNAs and miRNAs are a heterogeneous class of transcripts and are incompletely annotated.

In the present study, we performed microarray profiling analyses on differentially-expressed mRNAs and lncRNAs in CF and normal control rat hearts. Next, differentially-expressed mRNAs were applied to the Kyoto Encyclopedia of Genes and Genomes (KEGG) signaling annotation and Gene Ontology (GO) enrichment analyses to validate the essential roles of ECM and TSP1-enhanced TGFβ activation in CF. Co-expression network between differentially-expressed lncRNAs and ECM-related factors was constructed and lncRNA Homo sapiens ring finger protein 7 variant 5 and variant 6 (lnc RNF7), which was conservative in rat, mouse, and human, was selected and examined for its effects on CF *in vivo* and *in vitro*. Furthermore, miRNAs that might simultaneously target lnc RNF7 and TSP1 (THBS1) were identified and the predicted bindings between miR-543 and RNF7, between miR-543 and THBS1, were confirmed. Finally, the effects of the combination of lnc RNF7 and miR-543 on TSP1 and TGFβ activation were evaluated. In summary, we provided a novel mechanism of RNF7/miR-543 axis modulating CF in rats via TSP1-induced TGFβ activation.

## RESULTS

### The construction and identification of isoproterenol (ISP)-induced CF model in rats

**Figure 1:**
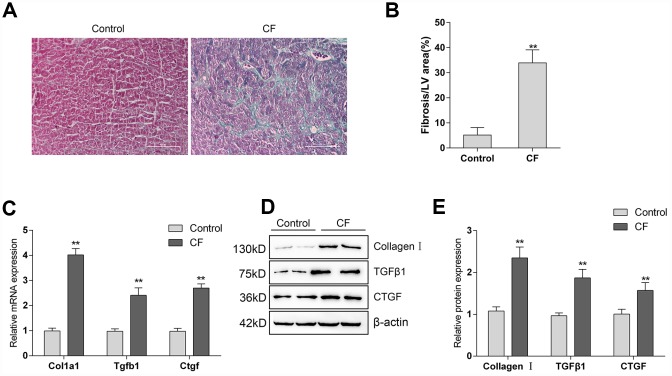
The construction and identification of ISP-induced cardiac fibrosis (CF) model in rats (**A**) Pathomorphological features of rat hearts in different groups examined by Masson staining. (**B**) In Masson staining, fibrotic tissue was stained to blue and myocardium to red. The fibrotic areas were calculated and the percentage of fibrotic tissue area was used to assess CF. (**C**–**E**) The mRNA expression and protein levels of Col1a1 (Collagen I), Tgfb1 (TGFβ1), and Ctgf (CTGF) in CF and control groups determined by real-time PCR and Immunoblotting analyses.

The rat CF model was first constructed via ISP induction. After induction, Masson staining and Immunoblotting assays were performed to validate the model. As represented by Masson staining, the fibrotic area was dramatically increased by ISP induction (Figure 1A, 1B). Moreover, the mRNA expression and protein levels of Collagen I and two potent fibrogenic factors, TGFβ1, and CTGF, were also detected. As shown in Figure 1C–1E, the mRNA expression and protein levels of Col1a1 (Collagen I), Tgfb1 (TGFβ1), and Ctgf (CTGF) were all significantly upregulated in ISP-induced rat CF model. These data indicate that the CF model in rats was successfully constructed.

### Differentially-expressed mRNAs in CF and normal rat hearts

**Figure 2:**
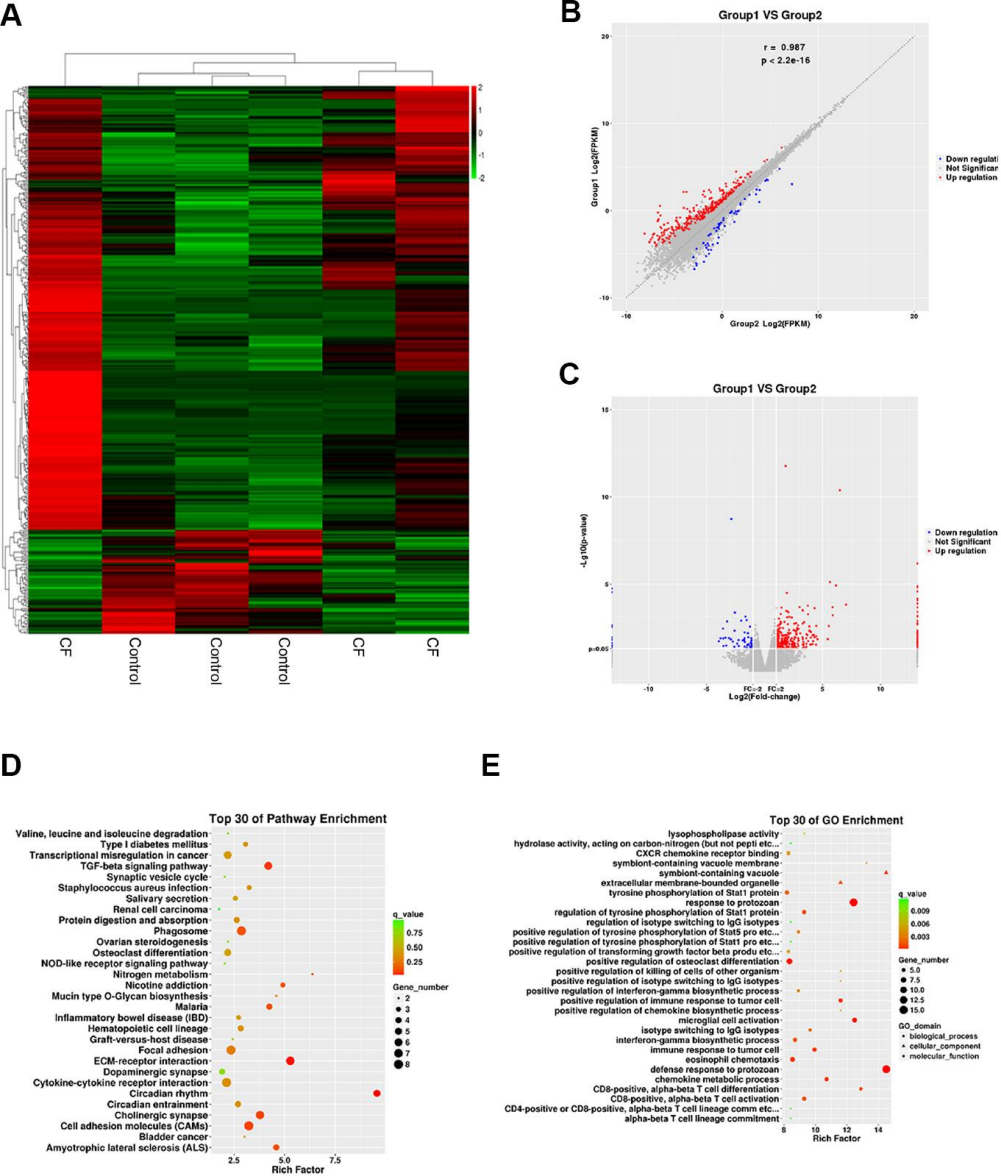
**Differentially-expressed mRNAs in CF and normal rat hearts.** (**A**) Hierarchical clustering of differentially-expressed mRNAs in CF and normal rat hearts. (**B**) Scatter diagram showing the expression correlation of these mRNAs. (**C**) Volcano plot diagram showing these differentially-expressed mRNAs. (**D**) KEGG signaling annotation on the differentially-expressed mRNAs. (**E**) GO Enrichment analyses on the differentially-expressed mRNAs.

To investigate the factors involved in the pathogenic processes, we performed microarray profiling analyses to identify differentially-expressed mRNAs and lncRNAs in CF and normal rat hearts. As represented by the hierarchical clustering diagram, a total of 55 mRNAs were significantly downregulated (Log2FC <= -0.56, p < 0.05) and 239 mRNAs were significantly upregulated (Log2FC>= 0.56, p < 0.05) (Figure 2A). These differentially-expressed mRNAs were also shown in the Scatter diagram (Figure 2B) and Volcano plot diagram (Figure 2C). Next, the differentially-expressed mRNAs were applied to the KEGG signaling annotation analyses. The results showed that these mRNAs were enriched in TGFβ signaling pathway (Rich factor = 5.28, p = 0.00018) and the ECM receptor interaction (Rich factor = 4.19, p = 0.0016), which both play a critical role in the fibrosis process (Figure 2D). Go enrichment analyses showed that differentially-expressed genes were concentrated in the regulation of cellular immunes, such as activation and maturation of CD8+ T cells (Figure 2E), suggesting that the extracellular microenvironment might be in an inflammatory state. Considering the essential roles of TGFβ-related inflammation and ECM in CF [10, 20], the study further conducted lncRNAs mining based on ECM and extracellular microenvironment alterations.

### Differentially-expressed lncRNAs in CF and normal rat hearts

**Figure 3:**
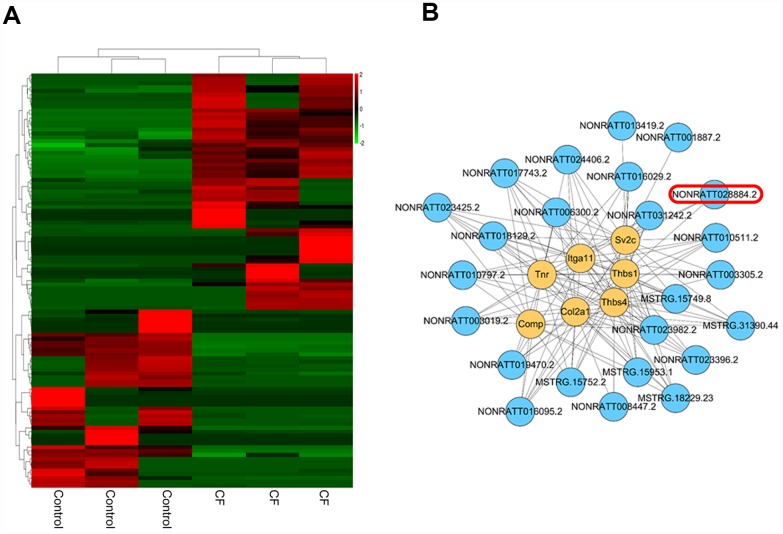
**Differentially-expressed lncRNAs in CF and normal rat hearts.** (**A**) Hierarchical clustering of differentially-expressed lncRNAs in CF and normal rat hearts. (**B**) Co-expression network (lncRNA/ECM receptor interaction) between differentially-expressed lncRNAs and extracellular matrix (ECM) receptors, including Itga11, Col2a1, Tnr, Thbs4, Thbs1, Sv2c, and Comp, was established by Pearson’s correlation analysis. (R > 0.8, p < 0.05).

The hierarchical clustering diagram showed that a total of 61 lncRNAs were significantly downregulated (Log2FC <= -0.56, p < 0.05) and 46 were upregulated (Log2FC>= 0.56, p < 0.05) (Figure 3A). Since CF is characterized by the alterations in ECM markers, next, a co-expression network was constructed between these differentially-expressed lncRNAs and several critical ECM markers, including Itga11, Col2a1, Tnr, Thbs4, Thbs1, Sv2c, and Comp. A total of 24 lncRNAs (MSTRG.15749.8, MSTRG.15752.2, MSTRG.15953.1, MSTRG.18229.23, MSTRG.31390.44, NONRATT001887.2, NONRATT003019.2, NONRATT003305.2, NONRATT006300.2, NONRATT008447.2, NONRATT010511.2, NONRATT010797.2, NONRATT013419.2, NONRATT016029.2, NONRATT016095.2, NONRATT017743.2, NONRATT018129.2, NONRATT019470.2, NONRATT023396.2, NONRATT023425.2, NONRATT023982.2, NONRATT024406.2, NONRATT028884.2, NONRATT031242.2) were significantly correlated with these ECM-related factors (*R* >0.8, *p* <0.05) (Figure 3B). Among them, NONRATT028884.2 was conservative in rat, mouse, and human (non-coding RNA, Homo sapiens ring finger protein 7 (RNF7), transcript variant 5 and 6); therefore, lnc RNF7 was selected for further experiments.

### Effect of lnc RNF7 silence on CF rat

**Figure 4:**
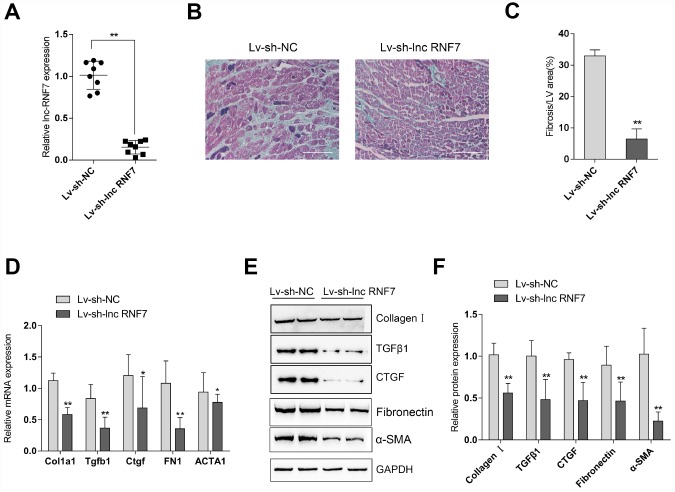
Effect of lnc RNF7 silence on CF rat (**A**) CF rats were injected with Lv-sh-lnc RNF7 or Lv-sh-NC and examined for the infection efficiency by real-time PCR. (**B**) Pathomorphological features of rat hearts in different groups examined by Masson staining. (**C**) The fibrotic areas were calculated and the percentage of fibrotic tissue area was used to assess CF. (**D**–**F**) The mRNA expression and protein levels of Col1a1 (Collagen I), Tgfb1 (TGFβ1), Ctgf (CTGF), FN1 (Fibronection), and ACTA1 (α-SMA) in CF and control groups determined by real-time PCR and Immunoblotting analyses.

To investigate the effects of lnc RNF7 on CF, CF rats were injected with Lv-sh-lnc RNF7 to achieve lnc RNF7 silence and examined by Masson staining, real-time PCR, and Immunoblotting. Lnc RNF7 silence was verified by real-time PCR (Figure 4A). Masson staining revealed that lnc RNF7 silence significantly reduced the fibrotic area in CF rats (Figure 4B, 4C). Consistently, lnc RNF7 silence also remarkably decreased the mRNA expression and protein levels of Col1a1 (Collagen I), Tgfb1 (TGFβ1), Ctgf (CTGF), FN1 (Fibronectin), and ACTA1 (α-SMA) (Figure 4D–4F). These data indicate that lnc RNF7 silence could significantly attenuate the inducible effects of ISP on rat CF.

### Effect of lnc RNF7 silence on primary rat cardiac fibroblasts proliferation and extracellular matrix deposition

**Figure 5:**
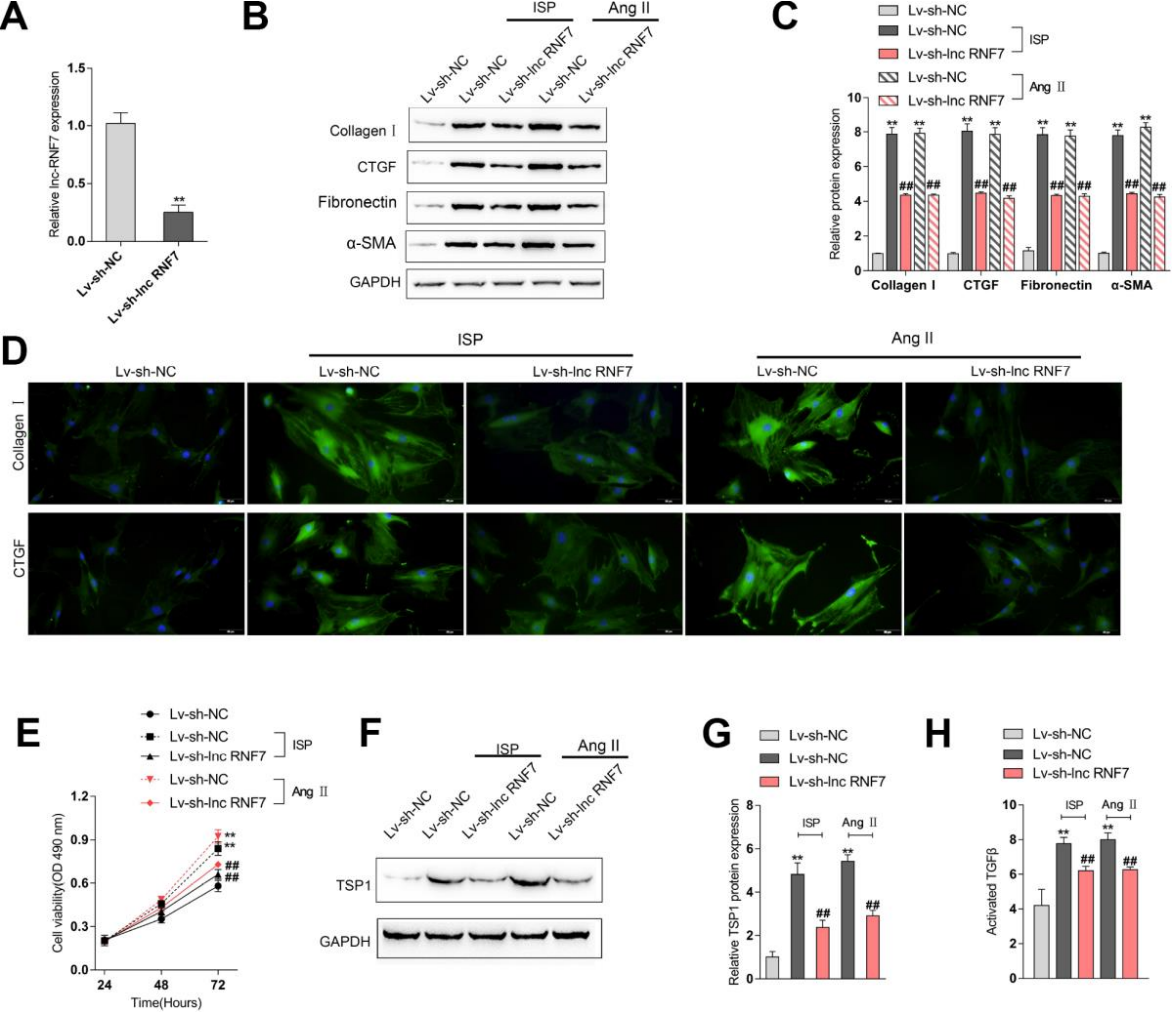
**Effect of RNF7 silence on primary rat cardiac fibroblasts proliferation and extracellular matrix deposition.** (**A**) Rat cardiac fibroblasts were infected with Lv-sh-lnc RNF7 or Lv-sh-NC and examined for the infection efficiency by real-time PCR. In the presence or absence of ISP or Ang II treatment, (**B**) the cellular content of Collagen I and CTGF were determined by IF staining. (**B**, **C**) The protein levels of Collagen I, CTGF, Fibronectin, and α-SMA were determined by Immunoblotting analyses. (**E**) The cell viability was determined by MTT assays. (**F**, **G**) The protein levels of TSP1 were determined by Immunoblotting analyses. (**H**) The TGFβ activity was determined by NRK colony formation assay.

To investigate the underlying mechanism of lnc RNF7 function on rat CF, we used ISP or Ang II to construct a fibrosis cell model on primary rat cardiac fibroblasts and then infected these cells with Lv-sh-lnc RNF7, as confirmed by real-time PCR (Figure 5A). Next, IF staining and Immunoblotting analyses revealed that the protein levels of ECM markers, Collagen I, CTGF, Fibronectin, and α-SMA were significantly increased by ISP or Ang II treatment while decreased by lnc RNF7 silence (Figure 5B–5D). Moreover, the proliferation of rat cardiac fibroblasts was significantly promoted by ISP or Ang II treatment while suppressed by lnc RNF7 silence (Figure 5E).

As we have mentioned, the matricellular protein TSP1 is a physiologic regulator of latent TGFβ activation *in vitro* and in many homeostatic and pathologic conditions *in vivo* [12–15]. Here, lnc RNF7 was significantly correlated with Thbs1 (TSP1); thus, we investigated the effect of RNF7 silence on TSP1 and TGFβ activity. In rat cardiac fibroblasts, ISP or Ang II treatment significantly increased the protein levels of TSP1, while ISP or Ang II-induced upregulation of TSP1 protein levels could be significantly reduced by lnc RNF7 silence (Figure 5F–5G). Consistently, ISP or Ang II treatment induced the activity of TGFβ, which could be significantly decreased by lnc RNF silence (Figure 5H). These data indicate that lnc RNF7 silence could attenuate ISP-induced fibrogenic changes in rat cardiac fibroblasts, most possibly via TSP1-regulated TGFβ activation.

### Selection and validation of miRNA that might target lncRNA RNF7 and THBS1

**Figure 6:**
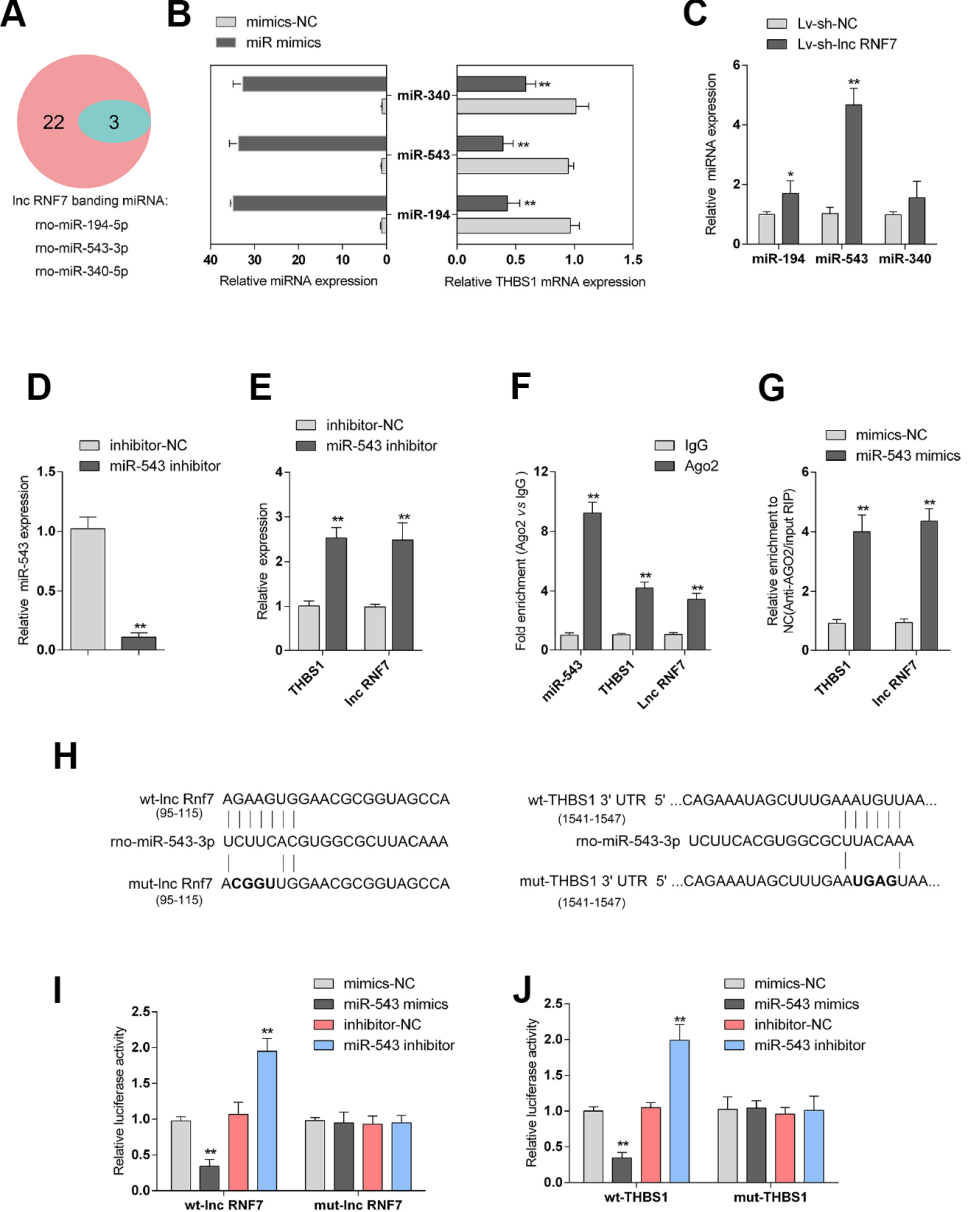
**Selection and validation of miRNA that might target lncRNA RNF7 and THBS1.** (**A**) A schematic diagram showing 25 miRNAs that might target THBS1 predicted by online tools; of them, 3 were predicted to target lncRNA RNF7. (**B**) The overexpression of these 3 miRNAs, miR-340, miR-543, and miR-194, was achieved in rat cardiac fibroblasts by transfection of miRNA mimics, as confirmed by real-time PCR. THBS1 mRNA expression in response to miRNA overexpression was determined by real-time PCR, respectively. (**C**) Rat cardiac fibroblasts were infected by Lv-sh-lnc RNF7 and examined for the expression of these miRNAs. (**D**) miR-543 inhibition achieved in rat cardiac fibroblasts by transfection of miR-543 inhibitor and confirmed by real-time PCR. (**E**) The expression of THBS1 and lnc-RNF7 in response to miR-543 inhibition was determined in cardiac fibroblasts by real-time PCR. (**F**) The levels of miR-543, THBS1, and lnc RNF7 precipitated by anti-AGO2 antibody were determined using RIP assays. (**G**) Endogenous THBS1 or lnc RNF7 pull-down by AGO2 upon overexpression of miR-543 was determined using RIP assays. (**H**) A schematic diagram showing the structures of wild- or mutant-type THBS1 3′-UTR or lnc RNF7 luciferase reporter vectors (wt-THBS1 3′-UTR/lnc RNF7 and mut-THBS1 3′-UTR/lnc RNF7). Mutant-type vectors contained a 4 bp mutation in the predicted miR-543 binding site. (**I**, **J**) These vectors were co-transfected into rat cardiac fibroblast with miR-543 mimics/inhibitor and the luciferase activity was determined.

LncRNAs act as endogenous sponges for other types of RNAs such as mRNAs and miRNAs [21–23]. Since RNF7 silence decreased the protein levels of TSP1, here, we hypothesize that RNF7 might affect TSP1 expression via miRNA. Online tools were employed to predict miRNAs that may target lnc RNF7 and THBS1 simultaneously, and a total of 3 miRNAs, miR-194, miR-543, and miR-340, were identified (Figure 6A). The overexpression of these miRNAs was achieved by transfection of miRNA mimics, as confirmed by real-time PCR (Figure 6B); the expression of THBS1 was significantly downregulated by these three miRNAs, more downregulated by miR-543 (Figure 6B). Moreover, after silencing lnc RNF7, the expression of the three miRNAs was significantly upregulated, and miR-543 was the most upregulated (Figure 6C). Therefore, miR-543 was selected for further experiments.

To further verify the prediction and selection of miR-543, we achieved miR-543 inhibition in rat cardiac fibroblasts by transfection of miR-543 inhibitor, as confirmed by real-time PCR (Figure 6D). In response to miR-543 inhibition, the expression of lnc RNF7 and THBS1 was both significantly upregulated (Figure 6E). Next, RNA binding protein Immunoprecipitation (RIP) assay and luciferase reporter assays were performed to validate the predicted bindings of miR-543 to lnc RNF7 and THBS1. As shown in Figure 6F, miR-543, lnc RNF7, and THBS1 were associated with the Argonaute 2 (AGO2) in rat cardiac fibroblasts. In RNA extracted from precipitated AGO2 protein, the levels of miR-543, lnc RNF7, and THBS1 were higher than those in the IgG group (Figure 6F). We also performed RIP assay in rat cardiac fibroblast transfected with control miRNA (mimics-NC) or miR-543 mimics followed by real-time PCR to detect lnc RNF7 and THBS1 associated with AGO2; the results shown in Figure 6G confirmed the interaction between lnc RNF7 and miR-543, and between THBS1 and miR-543. Furthermore, two types of luciferase reporter vectors, wild- and mutant-type lnc RNF7/THBS1 3′-UTR were constructed and named wt-lnc RNF7/THBS1 3′-UTR and mut-lnc RNF7/THBS1 3′-UTR. Mut-lnc RNF7/THBS1 3′-UTR contained a 4-bp mutation in the predicted miR-543 binding site (Figure 6H). These vectors were co-transfected into rat cardiac fibroblast with miR-543 mimics/inhibitor, and the luciferase activity was determined. As shown in Figure 6I and 6J, the luciferase activity was significantly suppressed by miR-543 overexpression while enhanced by miR-543 inhibition; after mutating the predicted miR-543 binding site, the changes in the luciferase activity were abolished. These data indicate that miR-543 could directly target RNF7 and 3′UTR of THBS1.

### Dynamic effects of lnc RNF7 and miR-543 on ECM and TGFβ activation via TSP1

**Figure 7:**
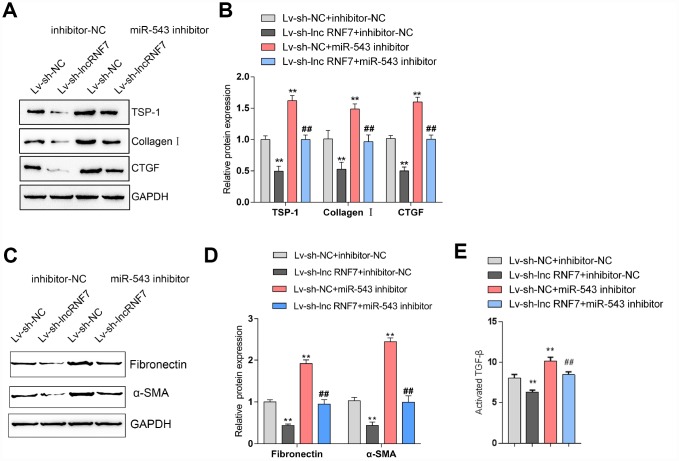
**Dynamic effects of lncRNA RNF7 and miR-543 on ECM and TGFβ activation via TSP1.** (**A**, **B**) Rat cardiac fibroblasts were co-transfected with miR-543 inhibitor and Lv-sh-lnc RNF7 and examined for the protein levels of TSP1, Collagen I, CTGF, Fibronectin, and (**C**)-SMA. (**C**) The TGFβ activity in co-transfected rat cardiac fibroblasts was determined by NRK colony formation assay.

As above-described, miR-543 targets lnc RNF7 and THBS1 and may be involved in the process of lnc RNF7 affecting rat CF via TSP1. Next, rat cardiac fibroblasts were co-transfected with miR-543 inhibitor and Lv-sh-lnc RNF7 and examined for the dynamic effects of lnc RNF7 and miR-543 on ECM-related factors and TGFβ activation in rat cardiac fibroblasts. Lnc RNF7 silence significantly decreased the protein levels of TSP1, Collagen I, and CTGF, while miR-543 inhibition exerted an opposing effect on these proteins; the effect of lnc RNF7 silence could be significantly reversed by miR-543 inhibition (Figure 7A, 7B). Consistently, lnc RNF7 silence suppressed, while miR-543 inhibition promoted the activity of TGFβ; the effect of lnc RNF7 silence was significantly reversed by miR-543 inhibition (Figure 7C). These findings indicate that lnc RNF7 could serve as a ceRNA for miR-543 to abolish miR-543-mediated THBS1 suppression, therefore modulating the ECM process via affecting TGFβ activation.

### The expression and correlation of miR-543 and THBS1 in rat hearts in control, Lv-sh-NC-, or Lv-sh-lnc RNF7-infection groups

**Figure 8:**
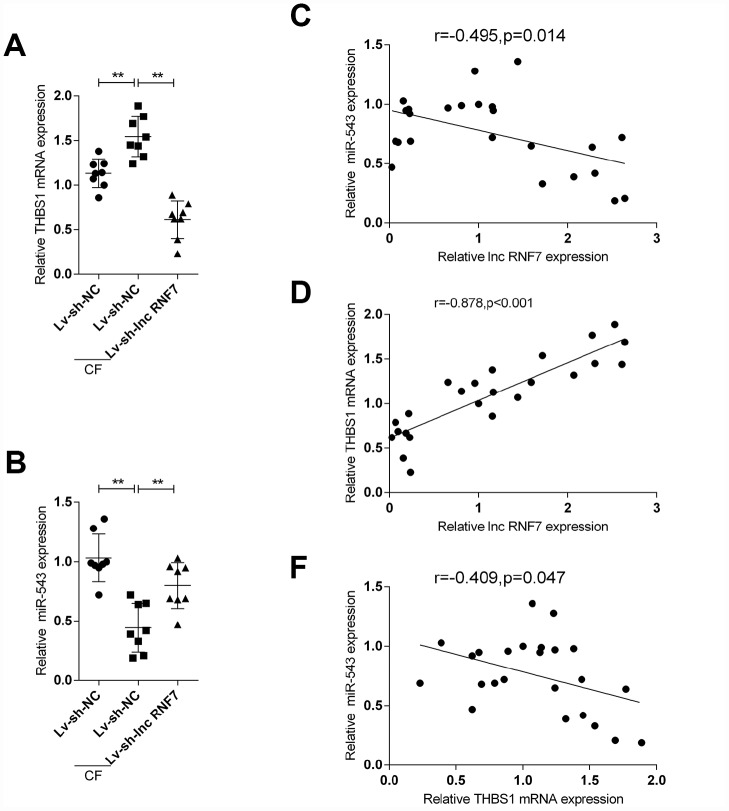
**The expression and correlation of miR-543 and THBS1 in rat hearts in control, Lv-sh-NC-, or Lv-sh-lnc RNF7-infection groups.** (**A**, **B**) Expression of miR-543 and THBS1 in rat hearts in control, Lv-sh-NC-, or Lv-sh-lnc RNF7-infection groups determined by real-time PCR. (**C**–**E**) Correlation of lncRNA RNF7, miR-543, and THBS1 in tissue samples analyzed by Pearman’s correlation analyses.

To further confirm the above findings, the expression of miR-543 and THBS1 in tissue samples and their correlations with lnc RNF7 were detected. The expression of THBS1 was significantly higher in the CF group compared to the normal group (Figure 8A); however, THBS1 expression was remarkably lower in lnc RNF7-silenced rat CF hearts compared to CF group (Figure 8A). On the contrary, miR-543 expression was downregulated in the CF group compared to the normal group (Figure 8B) while significantly rescued in lnc RNF7-silence rat CF hearts, compared to the CF group (Figure 8B). In tissue samples, miR-543 was negatively correlated with lnc RNF7 and THBS1, respectively (Figure 8C and 8E), while lnc RNF7 was positively correlated with THBS1 (Figure 8D).

## DISCUSSION

Herein, microarray profiling analysis revealed that a total of 294 mRNAs and 107 lncRNAs were differentially-expressed in CF rat hearts. The KEGG signaling annotation and GO enrichment analyses further confirmed the essential roles of ECM and TSP1-enhanced TGFβ activation in CF pathogenesis. Co-expression network construction and investigation revealed that lnc RNF7 was significantly correlated with TSP1 and ECM. Lnc RNF7 silence could attenuate ISP-induced CF in the rat heart *in vivo*. Lnc RNF7 silence could also mitigate ISP or Ang II-induced rat cardiac fibroblasts activation. Furthermore, as predicted by online tools and confirmed by RIP and luciferase reporter assays, miR-543 could simultaneously target lnc RNF7 and TSP1 (THBS1). Lnc RNF7 silence suppressed, while miR-543 inhibition promoted TSP1 protein and TSP1-mediated TGFβ activation, as well as ECM marker expression. The effects of lnc RNF7 silence could be significantly reversed by miR-543 inhibition.

In the remodeling, infarcted and pressure-overloaded myocardium TSP1 may modulate fibrotic responses by activating TGFβ [24] and by inhibiting matrix metalloproteinase (MMP) activity [25]. Gonzalez-Quesada el al. [26] demonstrated that db/db mice have increased TSP1 expression in the cardiac interstitium, associated with collagen deposition and fibrotic remodeling. Genetic disruption of TSP1 in db/db mice reduced collagen deposition while increased chamber dimensions, suggesting that the matrix-preserving actions of TSP1 may maintain chamber geometry [26]. In the present study, microarray profiling analyses identified a total of 294 differentially-expressed mRNAs in CF and normal rat hearts, most of which were enriched in TGFβ signaling pathway and the extracellular matrix (ECM) receptor interaction (Itga11, Col2a1, Tnr, Thbs4, Thbs1, Sv2c, Comp). These findings further yield the essential role of TSP1-enhanced TGFβ activation and ECM in CF pathogenesis, which was consistent with the studies mentioned above.

Non-coding transcripts are functionally active as physiological and pathological regulatory molecules in heart disease, including CF. For example, lncRNA CHRF was reported to regulate cardiac hypertrophy together with miR-489 and Myd88 [18]. Cardiac apoptosis-related lncRNA inhibits anoxia-induced mitochondrial fission and apoptosis in cardiomyocytes by impairing miR-539-dependent PHB2 downregulation [19], and MI-associated transcript promotes cardiac fibrosis by activating TGFβ1 [27]. MALAT1 has been reported to abolish the cardioprotective effects of Fentanyl [28]. Consistently, we also identified a total of 107 differentially-expressed lncRNAs in CF and normal rat hearts, and 24 of them were significantly correlated with ECM-related mRNAs (Itga11, Col2a1, Tnr, Thbs4, Thbs1, Sv2c, Comp), indicating that these lncRNAs may play a role in CF pathogenesis, possibly in TGFβ1 activation and ECM-related manners. Among these lncRNAs, lnc RNF7 was conservative in rats, mice, and humans. In the present study, lnc RNF7 silence significantly reduced the fibrogenic area in CF rat hearts and decreased the mRNA expression and protein levels Collagen I, TGFβ, and CTGF *in vivo*. As for the cellular functions, lnc RNF7 silence in rat cardiac fibroblasts significantly reduced ISP or Ang II-upregulated protein levels of Collagen I, TGFβ, and CTGF, the proliferation of rat cardiac fibroblasts, as well as TSP1 protein levels and TSP1-enhanced TGFβ1 activity *in vitro*. These data indicate that lnc RNF7 silence could improve ISP or Ang II-induced cardiac fibrosis in rats, *in vivo* and *in vitro*.

Concerning the molecular mechanism, lncRNAs act as endogenous sponges for other types of RNAs such as mRNAs and miRNAs [21–23]. It has become increasingly clear that numerous miRNA-binding sites exist on a wide variety of RNA transcripts, leading to the hypothesis that all RNA transcripts that contain miRNA-binding sites can communicate with and regulate each other by competing specifically for shared miRNAs, thus acting as ceRNAs [29–31]. By serving as ceRNAs for miRNAs, lncRNAs could counteract miRNA-mediated suppression on downstream target mRNAs. In the present study, online tools predicted that three miRNAs, miR-340, miR-543, and miR-194, could simultaneously target lnc RNF7 and the 3′UTR of THBS1 (TSP1). Of these three miRNAs, miR-543 was the most upregulated in response to lnc RNF7 silence. As further confirmed, miR-543 could directly target lnc RNF7 and THBS1; thus, lnc RNF7 might serve as a ceRNA for miR-543 to counteract miR-543-mediated suppression on THBS1, finally modulating CF progression in rats via ECM and TSP1-enhanced TGFβ activation. As expected, lnc RNF7 silence decreased, while miR-543 inhibition increased the protein levels of ECM markers, Collagen I, and CTGF, as well as TSP1 protein and TGFβ1 activity. More importantly, the effects of lnc RNF7 silence were significantly reversed by miR-543 inhibition, indicating that lnc RNF7/miR-543 axis modulates CF in rat cardiac fibroblasts via miR-543 downstream THBS1 and TSP1-mediated TGFβ1 activation.

Moreover, THBS1 mRNA expression was significantly upregulated in ISP-treated cardiac fibroblasts while downregulated in lnc RNF7-silenced cardiac fibroblasts upon ISP treatment. On the contrary, miR-543 expression was downregulated by ISP treatment while rescued by lnc RNF7 silence upon ISP treatment. In tissue samples, miR-543 was negatively correlated with lnc RNF7 and THBS1, respectively; lnc RNF7 and THBS1 were positively correlated with each other. We provided a solid experimental basis for a novel mechanism that the lnc RNF7/miR-543 axis regulates CF progression through ECM and TSP1-mediated TGFβ1 activation.

## MATERIALS AND METHODS

### Construction of the CF model in rats

Thirty adult male Sprague Dawley rats were bought from the Changsha SLAC Laboratory Animal Co. Ltd. Rats were subcutaneously injected with isoproterenol (ISP) (10 mg/kg) per day for 2 weeks, while rats were injected with an equal volume of saline solution as a control. The rats were sacrificed 30 days after injection.

For the identification of the CF model, rats were anesthetized with 10% chloral hydrate (300 mg/kg). Sternotomy was performed and the hearts were rapidly removed. Tissue from the left ventricle (LV) was rapidly frozen in liquid nitrogen and stored at -80°C for protein extraction, RNA isolation or Masson staining. The animal study was approved by the Animal Care Committee, Xiangya Hospital, Central South University.

### Knockdown of lnc RNF7 *in vivo* mediated by lentivirus

For the production of lentivirus, lnc RNF7 short hairpin RNA (sh-lnc RNF7) and negative control shRNA (sh-NC) were constructed into the lentiviral vector PHY-LV-KD5.1 (ThermoFisher Scientific, Waltham, MA) and then packed into lentivirus particles. Lentivirus particles were concentrated by centrifuge, and then 100 μl/heart (1 × 10^9^ TU/ml) lentivirus was injected into the left ventricular chamber. Fifteen minutes after injection, CF was induced.

### Microarray analyses on CF and normal heart tissues

Heart tissues from CF rats or normal control rats were selected for Next Generation Sequencing by illumine Hiseq 2000 (USA). To select the differentially expressed genes (mRNAs and lncRNAs), we used threshold values of |Log2FC| > 0.56 and a Benjamini–Hochberg corrected *P-*value of 0.05. The data were log2 transformed and median centered by genes using the Adjust Data function of CLUSTER 3.0 software (University of Tokyo, Human Genome Center, Tokyo, Japan) then further analyzed with hierarchical clustering with average linkage. Finally, these differentially-expressed genes were applied for KEGG signaling annotation (https://www.genome.jp/kegg/), and the visualization was achieved by using Cytoscape [32].

### Cell line, cell culture, and lentivirus infection

Primary rat cardiac fibroblasts were obtained from Procell (Wuhan, China). Cells were cultured in DMEM supplemented with 10% FBS in cell culture incubator (37 °C, CO_2_). Cells from earlier passages (2-4) were used. Cells were infected with lentivirus particles (Lv-sh-lnc RNF7 or Lv-sh-NC) in the presence of Polybrene (final concentration, 5 μg/ml). After 48 h, infection efficiency was examined by real-time PCR. For ISO or Ang II treatment, cardiac fibroblasts were treated with ISP (10 μM/L) or Ang II (1 μM/L) for 48 h. The cells were harvested for further experiments.

### Masson staining

After fixation for 24 h in 4% paraformaldehyde, the heart blocks were dehydrated, embedded in paraffin, and cut into 4-μm-thick slices. Slices were heated overnight at 37 °C, dewaxed, and stained with Masson trichrome using standard procedures [33, 34]. In Masson staining, fibrotic tissue was stained to blue and myocardium to red. The fibrotic areas were calculated with software Image-Pro (Meida Cybernetics, Bethesda, MD). The percentage of fibrotic tissue area was used to assess CF.

### Immunofluorescence (IF) staining

The cells were washed three times with PBS and fixed with 4% paraformaldehyde for 30 min at room temperature, then washed three times with PBS, followed by permeabilization with 0.5% Triton X-100 for 30 min at room temperature, and blocked with 1% bovine serum albumin (BSA)-supplemented PBS for 1 h and incubated overnight at 4°C or 4 h at 37°C with anti-Collagen I (ab34710, Abcam, Cambridge, MA, USA) and anti-CTGF (ab6992, Abcam). Later, the cells were labeled with appropriate fluorescein-labeled secondary antibody for 1 h in the dark at room temperature and followed by washing three times in PBS again (5 min each time). Meanwhile, the cell nuclei were counterstained with DAPI for 5 min, and then the samples were mounted on glass slides and examined on a fluorescence microscope.

### Cell viability determination by MTT analyses

A modified MTT assay was used to evaluate cell viability. 24 h after seeded into 96-well plates (5000 cells per well), cells treated with or without ISP or Ang II for 24, 48, and 72h, 20 μl MTT (at a concentration of 5 mg/ml; Sigma-Aldrich, St. Louis, MI, USA) was added, and the cells were incubated for an additional 4 h in a humidified incubator. After discarding the supernatant, 200 μl dimethyl sulfoxide was added to dissolve the formazan. OD_490 nm_ value was measured. The viability of the non-treatment cells (control) was defined as 100%, and the viability of cells from all other groups was calculated separately from that of the control group.

### TGFβ activity measurement

Culture supernatants were harvested and centrifuged at 1000 rpm for 10 min at 4 °C. Cell-free conditioned media were stored at -80 °C until used. TGFβ activity was performed using the NRK colony formation assay in soft agar, as previously described [35]. 0.5 ml conditioned media were directly added to each well. The plates were incubated for 7 days at the cell culture incubator. The number of colonies >62 mm in well was counted.

### Immunoblotting assays

Protein samples were separated by SDS-PAGE and transferred to nitrocellulose filters (Bio-Rad Laboratories, Hercules, CA, USA). Membranes were incubated overnight at 4*°*C with the appropriate antibodies and then washed and incubated for 1 h with horseradish peroxidase-conjugated goat anti-mouse or goat anti-rabbit IgG (Zymed, Waltham, MA, USA). The primary antibodies were as follows: anti-TGFβ1 (ab64715, Abcam), anti-TSP1 (ab1832, Abcam), anti-Collagen I (ab34710, Abcam), anti-CTGF (ab6992, Abcam), anti-fibronectin (15613-1-AP, Proteintech, USA), anti-α-SMA (14395-1-AP, Proteintech), and anti-GAPDH (ab8245, Abcam). Protein levels in each lane were normalized to the levels of GAPDH.

### RNA extraction and real-time PCR analysis

Total RNA was extracted from cultured cells using Trizol reagent (Invitrogen). A Hairpin-it TM miRNAs qPCR kit (Genepharma, Shanghai, China) was used to detect mature miRNA expression. The RNU6B expression was used as an endogenous control. The expression of mRNA was measured using an SYBR Green qPCR assay (Takara, Dalian, China). The expression of β-actin served as an endogenous control. The 2^-ΔΔCT^ method was applied for data processing.

### RNA immunoprecipitation (RIP)

RNA immunoprecipitation was performed using Magna RIP RNA-Binding Protein Immunoprecipitation Kit (17-700, Millipore) according to the manufacturer’s instructions. RNA for *in vitro* experiments was transcribed using T7 High YieldRNA Synthesis Kit (E2040S, NEB) according to the manufacturer’s instructions. IgG, miR-543, RNF7, and THBS1 levels in the immunoprecipitates were measured by qRT-PCR.

### Luciferase reporter assay

The fragment of lncRNA RNF7 or the 3′-untranslated region (3′-UTR) of THBS1 was amplified by PCR and cloned to the downstream of the Renilla psiCHECK2 vector (Promega, Madison, WI, USA), named wt-lnc RNF1 or wt-THBS1 3′-UTR. To generate the mutant reporter vectors, the study mutated the predicted miR-543 binding site in lncRNA RNF7 or THBS1 3′-UTR to remove the complementarity to miR-543 and named mut-lnc RNF1 or mut-THBS1 3′-UTR. Rat cardiac fibroblasts were co-transfected with the indicated vectors and miR-543 mimics/inhibitor, respectively. Luciferase activity was detected by the Dual-Luciferase Reporter Assay System (Promega, Fitchburg, WI, USA). Renilla luciferase activity was normalized to firefly luciferase activity for each transfected well.

### Statistical analysis

Data were exhibited as a mean ± SD of three independent experiments and processed using SPSS 17.0 statistical software (SPSS, Chicago, IL, USA). By using the Student’s test, we compared the differences between two groups. The differences among more than two groups were evaluated using the one-way ANOVA. *P* values of <0.05 were considered statistically significant.
