# Correction for: Long non-coding RNA RNF7 promotes the cardiac fibrosis in rat model via miR-543/THBS1 axis and TGFβ1 activation

**DOI:** 10.18632/aging.104222

**Published:** 2020-12-22

**Authors:** Fan Ouyang, Xiangyang Liu, Guoan Liu, Haihua Qiu, Yi He, Hongyu Hu, Ping Jiang

**Affiliations:** 1 Department of Cardiology, Zhuzhou Hospital, The Affiliated Hospital of Xiangya Medical College of Central South University, Changsha, Hunan, China

**Keywords:** Correction

**This article has been corrected:** The authors made a mistake in providing the wrong type of manuscript “Review” during the submission process. The correct type of this paper is “Research Paper”. This correction does not change the content of the publication.

Original article: Aging. 2020; 12:996–1010. . https://doi.org/10.18632/aging.102463

